# Choline as a Modulator of Periparturient Diseases in Dairy Cows

**DOI:** 10.3390/vetsci12101016

**Published:** 2025-10-21

**Authors:** Fenghong Wang, Yuanyin Guo, Xiu Su, Jie Cao

**Affiliations:** 1 Sano (Yangling) Modern Animal Nutrition Co., Ltd., Yangling 712199, China; fenghong121@163.com; 2 College of Animal Science and Technology, China Agricultural University, Beijing 100193, China; guoyuanyin979@163.com; 3 College of Veterinary Medicine, China Agricultural University, Beijing 100193, China; sy20243051209@cau.edu.cn

**Keywords:** dairy cow, periparturient disease, choline, regulatory mechanisms

## Abstract

**Simple Summary:**

During the periparturient period, dairy cows often face negative energy balance (NEB), which increases the risk of several metabolic and inflammatory diseases such as ketosis, fatty liver, mastitis, endometritis, and hypocalcemia. These disorders not only impair health but also reduce milk yield and reproductive performance. This article reviews that NEB disturbs lipid metabolism, triggers oxidative and endoplasmic reticulum (ER) stress, and weakens immune function. Choline, especially in the form of rumen-protected choline (RPC), helps alleviate these problems by improving liver fat metabolism, enhancing antioxidant capacity, and supporting immune defense. Supplementing RPC during the transition period can reduce the incidence of ketosis, fatty liver, and inflammation-related diseases, contributing to better health and productivity in dairy cows. This provides a reference for the mechanism of choline’s role in the pathogenic effects of NEB during the periparturient period.

**Abstract:**

Dairy cows experiencing negative energy balance (NEB) are prone to metabolic and inflammatory disorders, including ketosis, fatty liver, mastitis, endometritis, and hypocalcemia, which impair productive and reproductive performance. NEB elevates non-esterified fatty acids (NEFA) and β-hydroxybutyrate (BHBA), leading to disrupted lipid metabolism characterized by increased fatty acid synthesis (via SREBP-1c, ACC, FASN), impaired lipid export (downregulated MTTP, ApoB100, ACAT2), and reduced oxidation (suppressed SIRT1–PPARα–CPT1A/2 pathway), resulting in triacylglycerol (TAG) accumulation and ketosis. Excess reactive oxygen species (ROS) trigger oxidative and endoplasmic reticulum (ER) stress and apoptosis through JNK, p53/Nrf2, and PERK–eIF2α signaling, while HIF-2α–mediated hypoxia aggravates hepatic damage. Elevated NEFA/BHBA impair polymorphonuclear neutrophil (PMN) chemotaxis and phagocytosis, promoting mastitis and endometritis, and hypocalcemia further weakens immune defense. Rumen-protected choline (RPC) improves lipid metabolism by enhancing VLDL assembly and TAG export (upregulating MTTP, ApoB100, ATG3; inhibiting SREBF1, DGAT2), stimulating fatty acid oxidation (activating AMPK–PPARα–CPT1α), and reducing oxidative stress (suppressing ROS–ERN1). Moreover, RPC decreases IL-6 and TNF-α levels and enhances antioxidant capacity and PMN function. Overall, RPC alleviates NEB-induced metabolic and inflammatory diseases, supporting its inclusion in periparturient management to mitigate NEB and associated disorders.

## 1. Introduction

Choline (2-hydroxyethyl trimethylammonium) is a nutrient essential for normal liver, muscle, and brain function [1]. Choline was officially recognized as an essential nutrient by the U.S. Institute of Medicine in 1998 [2]. As an essential micronutrient, adequate choline intake in pregnant women reduces fetal brain developmental defects [3], cystic fibrosis, and hepatic steatosis [4]. Physiologically, choline is derived from both endogenous synthesis and exogenous dietary intake. Endogenously, choline is synthesized mainly in the liver [5] through the methylation of phosphatidylethanolamine (PE) to form phosphatidylcholine (PC) [6], and this choline accounts for about 95% of the total choline in mammalian tissues. However, the efficiency of this de novo synthesis pathway is limited and typically insufficient to meet a body’s full physiological demands [2], thus requiring dietary supplementation to maintain the internal choline homeostasis. While the daily choline requirements for various livestock and poultry species, such as chickens, pigs, and fish, have been determined, the specific dietary requirements for dairy cows remain unclear [7]. The current research on choline in non-ruminants, especially humans and rodents, is focused on its application during the perinatal period [8,9]. In these species, choline deficiency reduces PC synthesis and very-low-density lipoprotein (VLDL) secretion, leading to hepatic steatosis [10,11]. In contrast, ruminants have an inherently lower capacity for hepatic VLDL secretion [12], which significantly limits the triglyceride (TAG) export ability, thereby increasing the risk of hepatic fat accumulation during early lactation [13]. During the transition period, dairy cows experience substantial metabolic strain due to increased energy demands associated with lactation initiation, often leading to a negative energy balance (NEB) [14], which contributes to the development of fatty liver and also predisposes cows to a series of metabolic disorders, such as ketosis, abomasal displacement, mastitis, and retained placenta [15,16], severely compromising animal health and performance, particularly their lactation efficiency. Previous study have shown that supplementation of rumen-protected choline (RPC) can reduce hepatic TAG accumulation in dairy cows [17], thereby decreasing the incidence of fatty liver. RPC also lowers the risk of ketosis, ruminal acidosis, abomasal displacement, and mastitis [18,19] and improves production performance during the periparturient period [20]. However, the mechanisms underlying these effects of choline remain less explored, with no consolidated research reported in the literature. Therefore, this study aims to systematically review the pathogenic mechanisms and regulatory networks of the common periparturient diseases in dairy cows and explore the potential roles and targets of choline in these mechanisms, laying a theoretical foundation for the application of choline in disease prevention and health regulation during the periparturient period.

## 2. Molecular Mechanisms of Negative Energy Balance–Induced Diseases in Periparturient Dairy Cows

### 2.1. Ketosis and Fatty Liver

**Figure 1:**
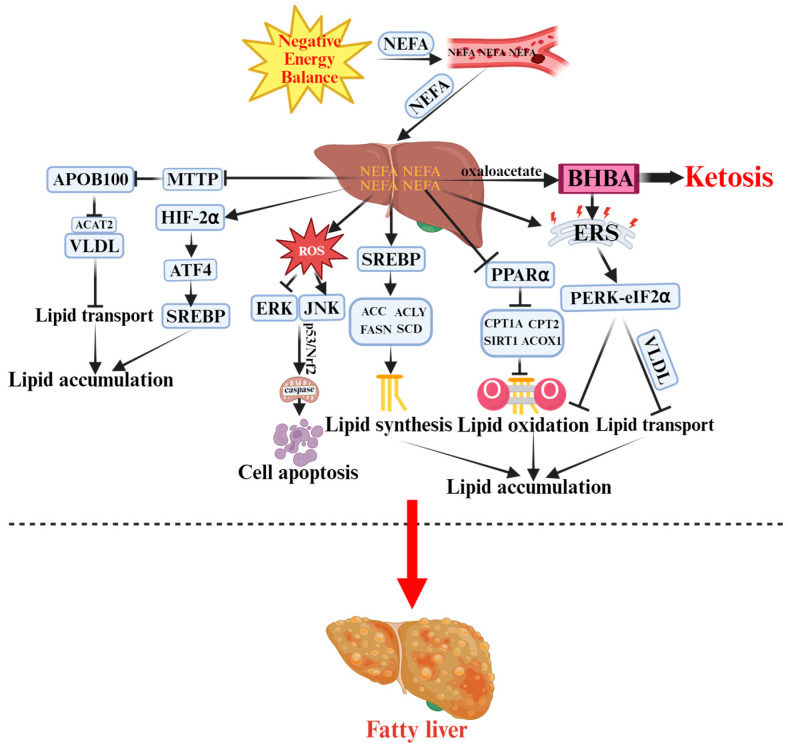
Pathogenesis of Ketosis and Fatty Liver in Periparturient Dairy Cows. 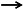 indicates a promoting effect or the direction of progression and 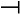 indicates an inhibitory effect. Negative energy balance (NEB) promotes the massive release of non-esterified fatty acids (NEFA) into the bloodstream, which are then transported to the liver. Incomplete oxidation of NEFA generates β-hydroxybutyrate (BHBA), triggering ketosis. Meanwhile, NEFA activates sterol regulatory element-binding protein (SREBP) to enhance lipid synthesis by upregulating acetyl-CoA carboxylase (ACC), ATP-citrate lyase (ACLY), and other enzymes. NEFA also inhibits microsomal triglyceride transfer protein (MTTP) and apolipoprotein B100 (ApoB100), reducing the export of very-low-density lipoproteins (VLDL). Additionally, NEFA suppresses the sirtuin 1 (SIRT1)–peroxisome proliferator-activated receptor alpha (PPARα)–carnitine palmitoyltransferase 1A/2 (CPT1A/2) pathway, decreasing fatty acid oxidation and promoting lipid accumulation. Excess reactive oxygen species (ROS) activate c-Jun N-terminal kinase (JNK) and extracellular signal-regulated kinase (ERK) signaling pathways. Protein kinase R-like endoplasmic reticulum kinase (PERK)–eukaryotic initiation factor 2α (eIF2α)–mediated endoplasmic reticulum stress (ERS) and hypoxia-inducible factor-2α (HIF-2α) signaling further exacerbate hepatic injury and apoptosis, ultimately leading to fatty liver.

Periparturient dairy cows frequently experience NEB, during which intensive lipomobilization releases large amounts of non-esterified fatty acids (NEFA) into the bloodstream [21]. A portion of these NEFA undergoes incomplete oxidation, leading to the accumulation of β-hydroxybutyrate (BHBA) and the onset of ketosis [22]. Figure 1 illustrates the pathogenesis of ketosis and fatty liver discussed in detail below. When NEB persists, excessive NEFA are esterified into triacylglycerol (TAG); however, due to the limited capacity for very low-density lipoprotein (VLDL) export, TAG accumulates within the liver, ultimately leading to hepatic steatosis [23]. The most prominent feature of fatty liver is enhanced hepatic lipogenesis, with fatty acid synthesis as the central process. Under NEB, elevated circulating NEFA further promote de novo lipogenesis. In vitro studies demonstrate that hepatocytes treated with exogenous NEFA show significantly increased protein levels of sterol regulatory element-binding protein-1c (SREBP-1c), acetyl-CoA carboxylase (ACC), fatty acid synthase (FASN), ATP-citrate lyase (ACLY), and stearoyl-CoA desaturase 1 (SCD1) [24]. As a key transcription factor, SREBP-1c regulates the expression of ACC, FASN, ACLY, and SCD1, thereby driving de novo fatty acid synthesis [25]. These findings indicate that high concentrations of NEFA may promote TAG synthesis through the upregulation of lipogenic genes, exacerbating hepatic lipid deposition. Hepatic lipid oxidation is critical for reducing lipid accumulation; however, when NEFA oxidation is insufficient, large quantities of BHBA are produced, further contributing to ketosis. During fatty acid oxidation, sirtuin 1 (SIRT1) modulates the activity of peroxisome proliferator-activated receptor alpha (PPARα) [26], which, in turn, enhances the expression of rate-limiting enzymes such as carnitine palmitoyltransferase 1A (CPT1A) and CPT2, thereby regulating mitochondrial fatty acid oxidation efficiency [27]. Additionally, acyl-CoA oxidase 1 (ACOX1), a key enzyme in peroxisomal β-oxidation, catalyzes the oxidation of long-chain fatty acids to pyruvate and acetyl-CoA [28]. Studies have demonstrated that moderate to high NEFA concentrations significantly downregulate PPARα and its target genes (CPT1A, CPT2, SIRT1, ACOX1) in bovine hepatocytes [29]. Moreover, SIRT1 not only enhances hepatic lipid oxidation but also inhibits lipogenesis [30]. Chamberlin et al. further revealed that NEFA may suppress the AMP-activated protein kinase (AMPK)/SIRT1 signaling pathway, thereby promoting lipid accumulation in bovine hepatocytes [31]. In addition to impaired oxidation, lipid transport dysfunction plays a decisive role in hepatic lipid accumulation. Hepatic lipid export depends on microsomal triglyceride transfer protein (MTTP), which facilitates lipid loading onto apolipoprotein B100 (ApoB100) to form VLDL for secretion into the bloodstream [32]. Acetyl-CoA acetyltransferase 2 (ACAT2), an essential component of both VLDL and LDL, also participates in hepatic TAG export [33]. High NEFA levels markedly suppress ApoB100, MTTP, and ACAT2 expression, reducing VLDL synthesis and assembly efficiency, and leading to intracellular TAG accumulation [34,35]. These findings collectively suggest that NEB-induced NEFA overload interferes with both hepatic lipid oxidation and transport, promoting hepatic steatosis. The core features of ketosis include systemic and hepatic oxidative stress, inflammation, and apoptosis, which are closely interconnected. In NEB-associated ketosis, excessive reactive oxygen species (ROS) generated during NEFA oxidation activates the c-Jun N-terminal kinase (JNK) pathway while inhibiting extracellular signal-regulated kinase (ERK). Through the Tumor protein p53/Nuclear factor erythroid 2–related factor 2 (p53/Nrf2) signaling axis and mitochondrial caspase cascades, these pathways induce hepatocellular apoptosis and lipotoxic injury [36]. Wen et al. further demonstrated that moderate to high NEFA concentrations trigger systemic inflammation via the Toll-like receptor 4/Myeloid differentiation primary response 88/Interleukin-1 receptor-associated kinase 2/Nuclear factor kappa-light-chain-enhancer of activated B cells (TLR4/MyD88/IRAK2/NF-κB) pathway, while NEFA-induced mitochondrial dysfunction amplifies lipid metabolism disorders and ROS generation, forming a feedback loop that exacerbates inflammation [37]. Endoplasmic reticulum (ER) stress also plays a pivotal role in the pathogenesis of ketosis and fatty liver. NEFA can directly induce ER stress, promoting lipid accumulation in calf hepatocytes [38], while BHBA similarly triggers ER stress responses [39]. Prolonged ER stress activates apoptotic pathways [40], aggravating liver injury and further advancing disease progression. Mechanistically, NEFA-induced ER stress primarily acts through the protein kinase R-like ER kinase (PERK)–eukaryotic initiation factor 2α (eIF2α) pathway, affecting lipid synthesis, oxidation, and VLDL secretion [41]. Furthermore, hepatic hypoxia during early lactation is another critical trigger of ketosis and fatty liver. Increased metabolic activity and oxygen consumption lead to hepatic hypoxic conditions [42]. Hypoxia-inducible factor 2α (HIF-2α), a classical regulator of hypoxia adaptation, contributes to hypoxia-induced lipogenesis [43]. In ketosis, NEFA promotes HIF-2α and its downstream target activating transcription factor 4 (ATF4), which subsequently enhances SREBP and its targets (FASN, ACACA, SCD), further promoting lipid deposition [44,45]. Beyond the liver, adipose tissue is another key metabolic organ in ketosis and fatty liver pathogenesis [46]. NEB promotes phosphorylation of perilipin 1 (PLIN1), facilitating its interaction with adipose triglyceride lipase (ATGL) and hormone-sensitive lipase (LIPE) to initiate lipolysis [47]. Lipolysis can activate autophagy to alleviate oxidative stress [48], but also promotes inflammation. In ketotic cows, lipolysis-derived ROS activate the NF-κB and NOD-like receptor family pyrin domain-containing 3 (NLRP3) pathways, upregulating Tumor Necrosis Factor-alpha and Interleukin-1 beta (TNF-α and IL-1β), forming a positive feedback loop between inflammation and lipolysis [49].

Collectively, NEB drives ketosis and fatty liver through coordinated disturbances in lipid metabolism (synthesis, oxidation, and transport), oxidative stress, inflammation, apoptosis, and ER stress, as well as hepatic hypoxia during early lactation. These interrelated mechanisms culminate in hepatic lipid accumulation and metabolic dysfunction, providing the molecular basis for periparturient metabolic disorders in dairy cows.

### 2.2. Mastitis

**Figure 2:**
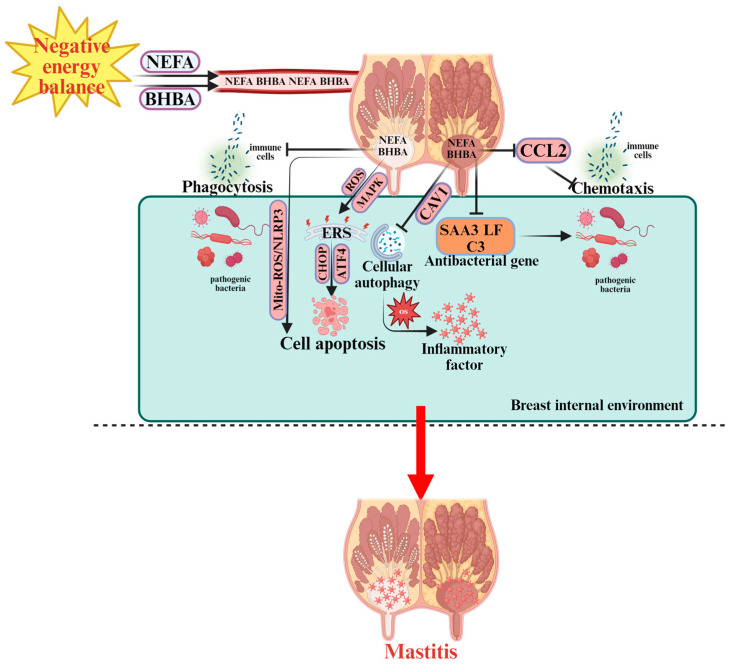
Pathogenesis of Mastitis in Periparturient Dairy Cows. 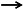 indicates a promoting effect or the direction of progression and 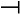 indicates an inhibitory effect. During NEB, non-esterified fatty acids (NEFA) and β-hydroxybutyrate (BHBA) are released into the bloodstream, affecting the breast internal environment. NEFA and BHBA disrupt immune cell function, including phagocytosis and chemotaxis, by downregulating C-C motif chemokine ligand 2 (CCL2), which impairs the immune response. The accumulation of NEFA and BHBA activates reactive oxygen species (ROS) and mitochondrial ROS (Mito-ROS), leading to oxidative stress (OS) and endoplasmic reticulum stress (ERS) via C/EBP homologous protein (CHOP), activating transcription factor 4 (ATF4), and other signaling pathways. This promotes cell apoptosis and cellular autophagy within immune cells. In response to bacterial infection, the activation of SAA3 (Serum Amyloid A3), lactoferrin (LF), and complement component C3 (C3) antibacterial genes aims to protect against pathogens. However, inflammatory factors are also produced, leading to further immune dysfunction and the onset of mastitis. The overall immune response is impaired by the altered immune cell function, ROS accumulation, and ER stress. These factors contribute to the progression of mastitis in dairy cows during NEB.

Negative energy balance (NEB) leads to immunosuppression in transition dairy cows. During NEB, the mobilization of body fat elevates plasma concentrations of NEFA and BHBA, which can reach the mammary gland via circulation. The rise in ketone bodies such as BHBA not only indicates a state of energy deficiency but also reflects potential impairment of immune function. Elevated BHBA levels adversely affect leukocyte chemotaxis, restricting their migration to infected mammary tissues. Consequently, cows in NEB exhibit reduced leukocyte recruitment to the mammary gland, severely weakening the local immune defense and increasing susceptibility to mastitis [50,51]. Figure 2 illustrates the pathogenesis of mastitis discussed in detail below. Under NEB conditions, leukocytes—particularly polymorphonuclear neutrophils (PMN) and macrophages—show impaired functionality, including decreased phagocytic activity against bacterial pathogens [50,51]. *Escherichia coli* is one of the predominant pathogens responsible for acute clinical mastitis in dairy cows [52]. Upon *Escherichia coli* (*E. coli*) invasion of mammary tissue, the host’s innate immune response is activated to combat infection. However, elevated BHBA concentrations significantly suppress this defense mechanism [53]. Specifically, BHBA reduces the expression of the chemokine gene C-C motif chemokine ligand 2 (CCL2), thereby impairing immune cell chemotaxis and infiltration into mammary tissue, and downregulates the expression of genes involved in humoral innate defense, including acute-phase protein Serum amyloid A3 (SAA3), antimicrobial peptide Lactoferrin (LF), and complement component 3 (C3). This multifaceted suppression diminishes the mammary gland’s ability to eliminate pathogens, rendering early-lactation cows more susceptible to mastitis. Following immunosuppression, bacteria penetrate the mammary gland through the teat canal, where Toll-like receptors (TLR2 and TLR4) recognize bacterial lipopolysaccharides (LPS) and trigger pro-inflammatory cytokine secretion [54]. Oxidative stress also contributes significantly to mastitis risk. Elevated NEFA concentrations induce systemic oxidative stress, damaging multiple tissues, including the mammary gland [55,56]. Reactive oxygen species (ROS) accumulate in mammary tissue via circulation, promoting local oxidative stress and upregulating pro-inflammatory mediators, ultimately leading to mastitis [57]. In vitro studies have shown that NEFA-induced oxidative stress is closely associated with autophagy [58,59]. Under clinical ketosis, high NEFA concentrations decrease caveolin-1 (CAV1) levels and inhibit autophagy, resulting in severe oxidative stress in mammary epithelial cells [60]. Moreover, oxidative stress can activate autophagy through the ROS–AMP-activated protein kinase-mechanistic target of rapamycin (AMPK–mTOR) signaling pathway, promoting Staphylococcus aureus invasion into host cells and contributing to mastitis development [61]. Thus, oxidative stress and autophagy are interdependent rather than unidirectional processes. Oxidative stress further activates the key inflammatory regulator NF-κB signaling pathway, driving inflammatory alterations in the mammary gland [55,62]. Among NEFA species, palmitic acid (PA)—the most abundant fatty acid during NEB [63]—induces ER stress and oxidative stress-mediated cell death in Mammary Alveolar Cells—Tumor line (MAC-T cells), a bovine mammary epithelial cell line [64]. Under ER stress, apoptosis is primarily mediated via the ATF4–C/EBP Homologous Protein (CHOP) signaling pathway [65], which is also linked to autophagy [66,67]. Additionally, NEFA can induce ER stress–mediated apoptosis in bovine mammary epithelial cells through the ROS–Mitogen-Activated Protein Kinase (MAPK) pathway [68]. Apoptosis of mammary epithelial cells is a central pathological event in mastitis and is regulated by NEB. Chang et al. demonstrated that NEFA induces apoptosis in mammary epithelial cells of ketotic cows via the mitochondrial ROS/NLRP3 inflammasome signaling pathway [69].

In summary, similar to the pathogenesis of ketosis and fatty liver, oxidative stress, ER stress, and apoptosis act in a coordinated manner rather than independently during the onset and progression of bovine mastitis. Through intricate crosstalk among these pathways—combined with immune suppression and dysregulated autophagy—NEB synergistically promotes mammary tissue damage, ultimately leading to mastitis in dairy cows.

### 2.3. Endometritis

**Figure 3:**
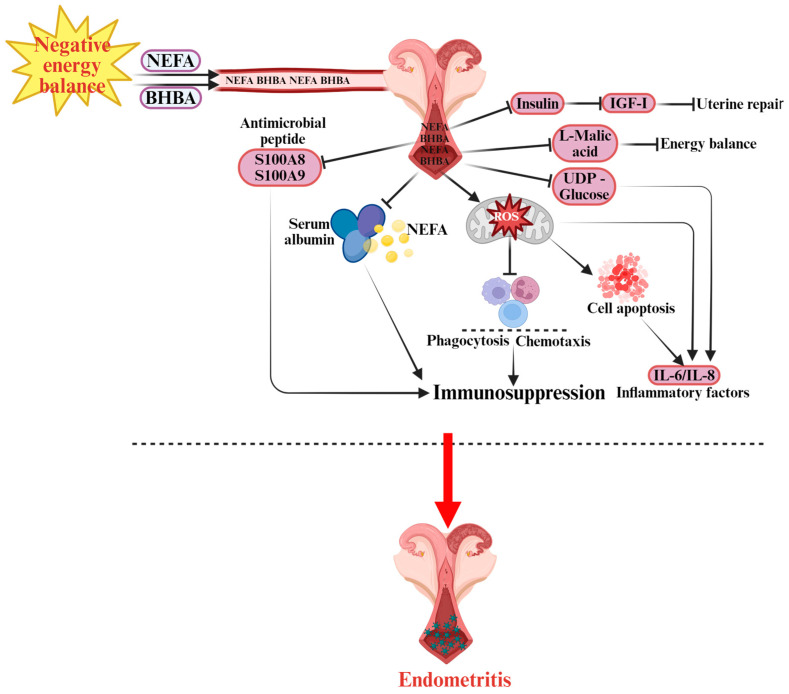
Pathogenesis of Endometritis in Dairy Cows. 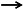 indicates a promoting effect or the direction of progression and 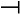 indicates an inhibitory effect. During NEB, non-esterified fatty acids (NEFA) and β-hydroxybutyrate (BHBA) are released into the bloodstream, affecting the uterine environment. The accumulation of NEFA impairs immune functions, including phagocytosis and chemotaxis, contributing to immunosuppression. Elevated levels of S100A8 (S100 calcium-binding protein A8) and S100A9 (S100 calcium-binding protein A9) antimicrobial peptides also disrupt immune defense. Additionally, serum albumin is involved in modulating lipid transport. Excessive reactive oxygen species (ROS) generated by NEFA negatively affect immune cells, leading to cell apoptosis and further weakening the immune response. The presence of insulin, insulin-like growth factor I (IGF-I), L-malic acid, and UDP-glucose (uridine diphosphate glucose) are related to energy balance and uterine repair processes. This dysfunction causes increased levels of inflammatory factors such as interleukin-6 (IL-6) and interleukin-8 (IL-8), which lead to the development of endometritis in dairy cows.

Negative energy balance (NEB) is a major predisposing factor for severe endometritis in dairy cows [70,71]. Evidence indicates that NEB markedly reduces the clearance capacity of the uterine endometrium and other mucosal tissues against free pathogens, thereby enhancing microbial invasion efficiency and amplifying systemic inflammatory responses [72]. These alterations are closely associated with metabolic disturbances and impaired immune cell function. The integrity of the immune barrier is strongly correlated with circulating free fatty acid levels [73], and cows affected by severe endometritis exhibit significantly higher plasma NEFA concentrations than healthy controls [74,75]. Figure 3 illustrates the pathogenesis of endometritis discussed in detail below. Excessive NEFA increases ROS production, which suppresses the activity of polymorphonuclear neutrophils (PMNs)—the most abundant immune cells in the endometrium, whose defensive roles mirror those in mastitis pathogenesis. Reduced PMN activity consequently compromises immune defense [76]. Concurrently, elevated serum BHBA levels are positively correlated with the incidence of Subclinical Endometritis (SE) [75,77]. High BHBA concentrations impair PMN chemotaxis and phagocytic capacity, ultimately inducing immunosuppression [78,79]. Cows with SE also exhibit reduced serum albumin concentrations [74], which are negatively correlated with elevated BHBA and NEFA levels [80]. The likely mechanism involves diminished NEFA-binding capacity of albumin, leading to an increase in free NEFA concentrations and exacerbation of immunosuppressive effects. Furthermore, Swangchan-Uthai et al. reported that NEB modulates the expression of local antimicrobial peptides S100 Calcium-binding Protein A8 and A9 (S100A8 and S100A9) in uterine epithelial tissue [81]. As crucial effectors of mucosal immunity, dysregulated expression of these peptides further weakens the local antimicrobial defense of the endometrium. Beyond immune modulation, NEB-induced metabolic disturbances contribute to altered carbohydrate metabolism. Key metabolites such as Uridine Diphosphate-glucose (UDP-glucose) (involved in inflammation regulation) and L-malate (associated with energy maintenance) are downregulated, resulting in weakened inflammatory regulation [82] and insufficient energy supply [83], thereby promoting the onset of endometritis. Inflammation and apoptosis are central mechanisms driving endometritis development [84]. In vitro studies show that NEFA promotes apoptosis of uterine epithelial cells and induces the release of pro-inflammatory cytokines interleukin-6 (IL-6) and interleukin-8 (IL-8) [85]. ROS accumulation due to oxidative stress further damages endometrial epithelial cells. Ferst et al. demonstrated that NEFA and BHBA elevate ROS levels, promote apoptosis, and impair endometrial cell function [86]. At the endocrine level, NEB inhibits insulin secretion and reduces growth hormone receptor expression, leading to decreased circulating insulin-like growth factor I (IGF-I) concentrations [87]. IGF-I is a key regulator of uterine tissue repair, and its deficiency markedly impairs tissue regeneration, increasing susceptibility to endometritis [88,89]. Beltman et al. further confirmed that plasma IGF-I not only reflects energy metabolism status but also serves as a potential biomarker for predicting endometritis risk [90].

In summary, NEB promotes the development of bovine endometritis through multiple synergistic mechanisms, including immunosuppression, oxidative stress, apoptosis, disruption of carbohydrate metabolism, and dysregulation of the insulin–IGF-I signaling pathway. Moreover, the pathogenic mechanisms underlying mastitis and endometritis share substantial similarities: in both diseases, NEB-induced inflammation and immune dysfunction constitute the core pathological basis. NEB triggers systemic inflammatory responses by impairing immune regulatory mechanisms in circulation. When the mammary gland or uterine endometrium is invaded by pathogenic bacteria, this compromised immune defense fails to mount an effective response, resulting in uncontrolled inflammation and tissue damage. Therefore, insights into NEB-mediated pathogenesis and control strategies for one condition can provide valuable reference for understanding and preventing the other.

To date, there is no direct evidence clarifying the precise molecular mechanism by which negative energy balance (NEB) contributes to hypocalcemia in dairy cows; however, studies have consistently shown that the incidence of hypocalcemia increases significantly during periods of NEB [91]. Notably, the intracellular regulation of calcium ions (Ca^2+^) appears to be closely linked to inflammatory responses and immunosuppression in dairy cows. Polymorphonuclear leukocytes (PMNLs) are critical immune effector cells in bovine defense mechanisms, and their activation relies on Ca^2+^ influx, which is essential for initiating and sustaining immune activity [92,93]. In cows affected by subclinical hypocalcemia, intracellular Ca^2+^ levels within PMNLs are markedly reduced. This deficiency directly impairs PMNL immune competence, manifesting as decreased cellular activity, diminished phagocytic capacity, and weakened migratory function [94]. Simultaneously, inadequate Ca^2+^ availability diminishes the generation of ROS [95]. The reduction in ROS not only weakens the host’s ability to eliminate pathogens but also disrupts normal inflammatory signaling [96]. Collectively, these dual effects—impaired immune cell activation and attenuated inflammatory signaling—significantly increase the risk of inflammation-related diseases such as mastitis and endometritis in dairy cows [97,98].

## 3. Effects of Choline on Negative Energy Balance–Induced Diseases in Periparturient Dairy Cows

### 3.1. Application of Choline in Periparturient Dairy Cow Diseases

Once periparturient diseases progress in severity, they can significantly impair both the productive and reproductive performance of dairy cows, ultimately reducing the overall economic efficiency of dairy operations. Productive performance is primarily reflected in lactation traits, including milk yield and milk composition (fat, protein, and lactose content). Dry matter intake (DMI) is also a critical indicator of productive efficiency in periparturient cows. Reproductive performance, on the other hand, is directly related to the continuous supply and replacement efficiency of breeding females and indirectly influences the profitability and sustainability of dairy herds. Nearly all studies have employed RPC supplementation, as the choline ingested by ruminants is rapidly degraded by the microorganisms present in the rumen [99]. As early as the 1980s, it was reported that both abomasal infusion and dietary supplementation of choline could significantly increase milk yield and milk fat percentage in dairy cows [100,101]. In specific application studies of RPC, different supplementation regimens have shown different effects. Supplementing 12.9 g/d RPC significantly increased both prepartum and post-partum DMI, along with increased milk yield, energy-corrected milk (ECM) production, milk fat percentage, and milk protein percentage [19]. When 15 g/d or 22 g/d RPC is fed, it improved colostrum yield without affecting colostrum quality and that Energy-Corrected Milk (ECM) production tended to increase during the first three weeks post-partum. In addition, milk fat and protein yields and the milk protein content also increased, although prepartum DMI was significantly reduced, which was attributed to a satiety effect induced by RPC supplementation [102]. The improvement in the milk protein content was attributed to the methyl donor function of RPC and its methionine-sparing effect [103]. The mechanisms through which RPC increases milk yield during the periparturient period may involve its lipotropic effect on enhancing hepatic fat export [104] and its function as a methyl donor that increases the methionine concentrations [105]; in cases of methionine deficiency, RPC supplementation reportedly improved milk yield [106]. Furthermore, RPC may boost the utilization of dietary or mobilized fatty acids by the mammary gland, thereby increasing milk fat synthesis [107]. Similarly, study on dairy goats have also observed that RPC significantly increased milk fat and yield [108]. Discrepancies exists in findings regarding DMI. Bollatti et al. reported that supplementing 60 g/d RPC throughout the periparturient period exerted no effect on post-partum DMI [20], which may be attributed to the differences in the methionine concentrations in the diet, as the efficacy of RPC in dairy cows appears to be negatively correlated to the dietary methionine levels [19].

In addition to improving productive performance, RPC has also been applied to enhance reproductive performance in dairy cows. Studies have shown that feeding 10 g/d of RPC to dairy cows starting from 20 days prepartum and continuing for 30 days can promoted an earlier peak in P4 concentration after calving, advanced the time to first service by about 7.3 days, and shortened the calving interval by nearly 19.4 days; however, the conception rate decreased [109]. P4 is a key physiological indicator for assessing reproductive status during early lactation and can reflect the conception status at first service, the number of inseminations, and the days open [110]. The trend of P4 levels is often used to determine the degree of recovery of reproductive function [111]. However, another study have reported that RPC has no significant effect on the P4 concentration [18]. In terms of other reproductive indicators, supplementing Holstein dairy cows with 60 g/d of RPC significantly reduced the average number of inseminations per conception and days open but exerted no significant effect on the time to first estrus or the number of pregnant cows [112]. Overall, RPC appears to be beneficial for improving reproductive performance in dairy cows. However, the results for reproductive parameters remain inconsistent. Morrison et al. reported that supplementing cows with 100 g/d RPC starting 21 days before calving exerted no significant effects on the incidence of anovulation within 8 weeks post-partum, the pregnancy rate at first service, or the median days to pregnancy within 200 days post-partum [113]. Similarly, Acosta et al. reported no significant improvements in reproductive indicators, such as the days to first ovulatory estrus, growth rate, or diameter of dominant follicles, when multiparous Holstein cows were supplemented with 60 g/d of choline starting 21 days before calving [114]. In summary, choline can improve the productive performance of dairy cows by increasing DMI, enhancing milk yield, and optimizing milk composition, particularly milk fat and protein contents. Moreover, it exerts positive effects on reproductive performance by regulating progesterone levels, shortening the calving interval, reducing the number of inseminations per conception, and decreasing days open. Beyond its role in improving productivity and reproduction impaired by periparturient metabolic disorders, existing research also demonstrates that choline supplementation effectively reduces the incidence of major periparturient diseases in dairy cows. Epidemiological data indicate that the prevalence of ketosis in dairy herds is approximately 15%, exceeding the epidemiological threshold of 10% [115]; the global incidence of mastitis ranges between 20% and 30%; although data on endometritis are relatively limited, recent studies from Chinese dairy farms report a prevalence of about 10.68% [116]. For hypocalcemia, the clinical form occurs in fewer than 3% of cows, but the global prevalence of subclinical hypocalcemia can reach as high as 60–80% [117], posing a much greater latent risk than clinically apparent cases. Supplementation with rumen-protected choline (RPC) from 21 days before to 21 days after calving has been shown to reduce the incidence of subclinical ketosis from 12.7% to 4.2% [118] and significantly lower the risk of mastitis [19]. During the same period, daily supplementation with 60 g RPC effectively decreased the occurrence of subclinical endometritis [119], while providing 12.9 g of choline ions per day in RPC form reduced the incidence of subclinical hypocalcemia in multiparous cows by 20.9% [120].

### 3.2. Mechanisms of Choline Action in Negative Energy Balance–Induced Diseases of Periparturient Dairy Cows

**Figure 4:**
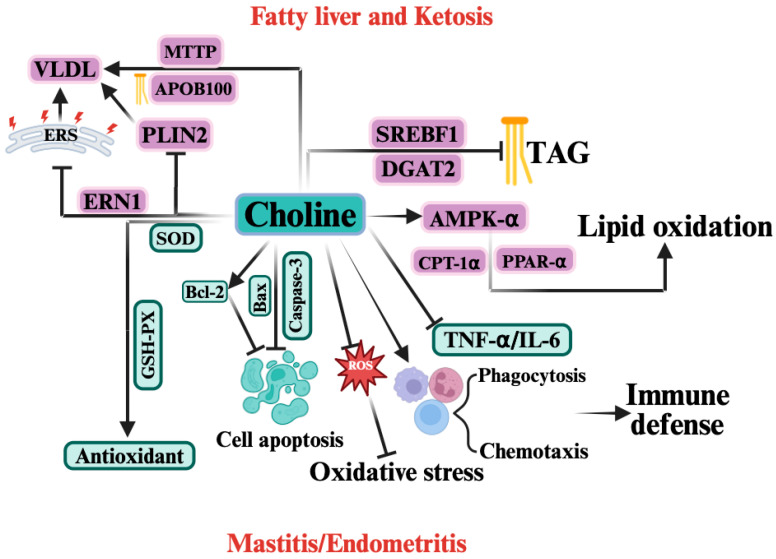
The Mechanism of Choline in Periparturient Diseases Induced by Negative Energy Balance (NEB). 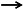 indicates a promoting effect or the direction of progression and 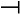 indicates an inhibitory effect. In the context of NEB, choline exerts protective effects by regulating multiple biological pathways. Choline enhances lipid oxidation by activating AMP-activated protein kinase (AMPK-α), peroxisome proliferator-activated receptor α (PPAR-α), and carnitine palmitoyltransferase 1α (CPT-1α), which helps reduce triacylglycerol (TAG) accumulation and improve fatty acid oxidation. Choline also upregulates microsomal triglyceride transfer protein (MTTP) and apolipoprotein B100 (ApoB100), facilitating the assembly and export of very low-density lipoprotein (VLDL). Additionally, it reduces lipid accumulation through inhibition of sterol regulatory element-binding protein (SREBF1) and diacylglycerol acyltransferase 2 (DGAT2). Choline also acts as an antioxidant, enhancing the activity of superoxide dismutase (SOD) and glutathione peroxidase (GSH-Px), thereby mitigating oxidative stress. It also reduces reactive oxygen species (ROS) production and regulates cell apoptosis by modulating key apoptotic proteins, including B-cell lymphoma 2 (Bcl-2), Bax, and caspase-3. Furthermore, choline improves immune defense by enhancing phagocytosis and chemotaxis of immune cells and reducing inflammatory cytokines such as tumor necrosis factor alpha (TNF-α) and interleukin-6 (IL-6). Collectively, these actions help alleviate mastitis and endometritis associated with NEB.

At present, research investigating whether choline can effectively alleviate NEB in dairy cows remains limited, and the available evidence is still insufficient. Therefore, this section focuses on elucidating the roles and underlying mechanisms of choline in modulating the development of periparturient diseases induced by NEB in dairy cows. Figure 4 illustrates the mechanism by which choline acts in periparturient diseases induced by negative energy balance (NEB), which is discussed in detail below. In relation to ketosis and fatty liver, regarding hepatic lipid transport, supplementation with RPC can promote hepatic TAG export by upregulating the expression of MTTP and APOB100 [121], thereby reducing hepatic triacylglycerol accumulation, but has no effect on blood NEFA and BHBA levels in dairy cows [122]. Previous study have also reported that supplementation with 12.9 g of choline ions could inhibit hepatic lipid deposition without significantly altering the expression of lipolysis-related genes [121], suggesting that RPC promotes TAG efflux primarily by enhancing VLDL synthesis rather than inhibiting fat mobilization. Moreover, autophagy is important for lipid droplet degradation and can effectively reduce hepatic lipid accumulation [123,124]. RPC may promote the conversion and export of intrahepatic lipid droplets to VLDL by reducing the stabilizing effect of PLIN2 and increase the expression of the autophagy gene Autophagy-related gene 3 (ATG3) [125]. However, one study has shown that RPC supplementation (5.6–25.2 g/d) does not significantly reduce hepatic TAG synthesis [19], probably because choline is extensively secreted into milk during early lactation [126,127], which weakens its regulatory effects on the liver. These findings suggest that a higher dose of RPC may be required during early lactation to achieve a reduction in hepatic lipids. In terms of lipid synthesis and oxidation, RPC supplementation can significantly inhibits the expressions of Diacylglycerol O-Acyltransferase 2 (DGAT2) and SREBF1 [128]. In NEFA- or BHBA-induced hepatocyte injury models, choline supplementation could increase fatty acid oxidation and transport in NEFA-treated hepatocytes by promoting the phosphorylation of AMPK-α, upregulating PPAR-α, CPT-1α, and downregulating SREBP-1c expressions [129]. Regarding oxidative stress and endoplasmic reticulum stress, RPC can also stabilize mitochondrial membrane structure, reduce ROS release to alleviate oxidative stress [130], and downregulate Endoplasmic Reticulum to Nucleus Signaling 1 (ERN1) expression to relieve endoplasmic reticulum stress, thereby promoting VLDL synthesis and TAG export [122]. In relation to hypoxia, although direct evidence for the relationship between choline and HIF-2α in dairy cows is currently lacking, studies on mice have shown that choline deficiency can induce HIF-2α activation in hepatocytes, thereby contributing to the emergence and progression of fatty liver [131], which indirectly suggests that choline may alleviate the hepatic metabolic burden by inhibiting HIF-2α. However, given the complexity of the mechanisms underlying ketosis and fatty liver, the regulatory role of choline requires further validation. Therefore, choline may alleviate the occurrence and progression of ketosis and fatty liver under negative energy balance by promoting hepatic lipid transport and oxidation, while reducing hepatic oxidative stress, endoplasmic reticulum stress, and lipid synthesis.

Inflammation and the associated inflammatory cytokines represent common pathological features of both mastitis and endometritis. Previous studies have shown that supplementation with RPC exerts beneficial effects in mitigating inflammatory responses [122]. Periparturient supplementation with RPC has been reported to reduce circulating concentrations of IL-6 and TNF-α in dairy cows [132]. Regarding immune suppression, supplementation with RPC from 17 ± 4.6 days before calving to 21 days postpartum enhances innate cellular immunity by increasing the proportion of circulating neutrophils involved in phagocytosis and oxidative burst [133]. Similarly, Lopreiato et al. confirmed that choline significantly enhances the overall activation of polymorphonuclear leukocytes [134]. Oxidative stress is a shared etiological factor underlying ketosis, fatty liver, mastitis, and endometritis. During hepatic metabolism, elevated NEFA levels promote the excessive production of ROS, which not only induce oxidative stress in the liver but also circulate to peripheral tissues such as the mammary gland and endometrium. In the mammary gland, ROS accumulation enhances pro-inflammatory cytokine expression, thereby contributing to mastitis development [57]. Similarly, ROS accumulation in the endometrium promotes oxidative stress–induced inflammatory injury. RPC can stabilize mitochondrial membrane integrity, reducing ROS release and thereby alleviating oxidative stress. In addition, RPC supplementation significantly increases serum catalase levels from 10 days prepartum to 20 days postpartum, as well as superoxide dismutase levels at calving and glutathione peroxidase (GSH-Px) levels postpartum, indicating that RPC enhances the antioxidant defense system and mitigates oxidative stress [135]. With respect to apoptosis, in bovine mammary epithelial cell models, choline supplementation effectively downregulates caspase-3 and Bcl-2-associated X protein (Bax) expression while upregulating B-cell lymphoma 2 (Bcl-2), suggesting an anti-apoptotic role. This effect is likely mediated through modulation of the PERK/Nrf2 signaling pathway [136]. However, the potential anti-apoptotic effects of choline on endometrial epithelial cells remain unknown. In summary, choline may alleviate NEB-induced mastitis and endometritis by enhancing immune function, exerting anti-apoptotic activity, and suppressing oxidative stress, endoplasmic reticulum stress, and inflammatory cytokine production.

## 4. Conclusions

In the pathogenesis of ketosis and fatty liver, negative energy balance (NEB) primarily acts through four major pathways: disruption of lipid metabolism, induction of oxidative stress, activation of inflammatory responses, and regulation of apoptosis and ER stress. These alterations—characterized by enhanced lipid synthesis, impaired lipid transport, and suppressed lipid oxidation—collectively lead to hepatic triglyceride accumulation, establishing the pathological basis of both disorders. In contrast, NEB contributes to mastitis and endometritis mainly by inducing immunosuppression, thereby weakening host defense, promoting oxidative and ER stress, and triggering abnormal apoptosis that facilitates pathogen invasion and inflammation spread. Moreover, dysregulated calcium homeostasis in hypocalcemia appears to be associated with these inflammatory diseases, underscoring the interconnection between metabolic and immune dysfunctions in periparturient cows. Nutritionally, choline supplementation has been shown to reduce the incidence of ketosis, fatty liver, mastitis, endometritis, and hypocalcemia, with disease-specific mechanisms. In metabolic disorders, choline enhances hepatic lipid oxidation and VLDL export, suppresses excessive lipogenesis, and alleviates hepatic oxidative and ER stress. In inflammation-related diseases, choline improves immune function, enhances cellular anti-apoptotic capacity, and suppresses oxidative stress, ER stress, and pro-inflammatory cytokine release. Although evidence directly linking NEB to hypocalcemia remains limited, the potential relationship warrants further study. Likewise, the regulatory pathways through which choline mitigates NEB-induced disorders only partially overlap with NEB-driven mechanisms, highlighting the need for additional in vivo and in vitro research to clarify their distinct and shared molecular targets.

## Data Availability

No new date were created or analyzed in this study. Date sharing is not applicable to this article.

## Abbreviations

The following abbreviations are used in this manuscript:

ACAT2	Acetyl-CoA acetyltransferase 2
ACC	Acetyl-CoA carboxylase
ACLY	ATP-citrate lyase
ACOX1	Acyl-CoA oxidase 1
AMPK	AMP-activated protein kinase
AMPK–mTOR	AMP-activated protein kinase–mechanistic target of rapamycin
APOB100	Apolipoprotein B100
ATF4	Activating transcription factor 4
ATG3	Autophagy-related gene 3
ATGL	Adipose triglyceride lipase
Bax	Bcl-2-associated X protein
Bcl-2	B-cell lymphoma 2
BHBA	β-Hydroxybutyrate
CAV1	Caveolin-1
Ca^2+^	Calcium ions
CCL2	C-C motif chemokine ligand 2
CHOP	C/EBP homologous protein
CPT1A	Carnitine palmitoyltransferase 1A
CPT2	Carnitine palmitoyltransferase 2
DMI	Dry matter intake
*E. coli*	*Escherichia coli*
ECM	Energy-corrected milk
EIF2α	Eukaryotic initiation factor 2α
ER	Endoplasmic reticulum
ERK	Extracellular signal-regulated kinase
ERN1	Endoplasmic reticulum to nucleus signaling 1
FASN	Fatty acid synthase
GSH-Px	glutathione peroxidase
HIF-2α	Hypoxia-inducible factor 2α
IGF-I	Insulin-like growth factor I
IL-6 and IL-8	Interleukin-6 and Interleukin-8
JNK	c-Jun N-terminal kinase
LF	Lactoferrin
LIPE	Hormone-sensitive lipase
LPS	Lipopolysaccharides
MAC-T cells	Mammary alveolar cells—tumor line
MAPK	Mitogen-activated protein kinase
MTTP	Microsomal triglyceride transfer protein
NEB	Negative energy balance
NEFA	Non-esterified fatty acids
NLRP3	NOD-like receptor family pyrin domain-containing 3
PA	Palmitic acid
PC	Phosphatidylcholine
PE	Phosphatidylethanolamine
PERK	Protein kinase R-like ER kinase
PLIN1	Perilipin 1
PMNLs	Polymorphonuclear leukocytes
PMNs	Polymorphonuclear neutrophils
PPARα	Peroxisome proliferator-activated receptor alpha
ROS	Reactive oxygen species
RPC	Rumen-protected choline
SAA3	Serum amyloid A3
SCD1	Stearoyl-CoA desaturase 1
SE	Subclinical endometritis
SIRT1	Sirtuin 1
SREBP-1c	Sterol regulatory element-binding protein-1c
S100A8 and S100A9	S100 calcium-binding protein A8 and A9
TAG	Triglyceride
TLR4/MyD88/IRAK2/NF-κB	Toll-like receptor 4/Myeloid differentiation primary response 88/Interleukin-1 receptor-associated kinase 2/Nuclear factor kappa-light-chain-enhancer of activated B cells
TNF-α and IL-1β	Tumor necrosis factor-alpha and interleukin-1 beta
UDP-glucose	Uridine diphosphate-glucose
VLDL	Very-low-density lipoprotein

## References

[1] Bernhard W., Lange R., Graepler-Mainka U., Engel C., Machann J., Hund V., Shunova A., Hector A., Riethmüller J. (2019). Choline supplementation in cystic fibrosis—The metabolic and clinical impact. Nutrients.

[2] Subcommittee on Upper Reference Levels of Nutrients, Standing Committee on the Scientific Evaluation of Dietary Reference Intakes and its Panel on Folate, Other B Vitamins and Choline (2000). Dietary Reference Intakes for Thiamin, Riboflavin, Niacin, Vitamin B6, Folate, Vitamin B12, Pantothenic Acid, Biotin, and Choline.

[3] Freedman R., Hunter S.K., Law A.J., Clark A.M., Roberts A., Hoffman M.C. (2022). Choline, folic acid, Vitamin D, and fetal brain development in the psychosis spectrum. Schizophr. Res..

[4] Buchman A.L., Ament M.E., Sohel M., Dubin M., Jenden D.J., Roch M., Pownall H., Farley W., Awal M., Ahn C. (2001). Choline deficiency causes reversible hepatic abnormalities in patients receiving parenteral nutrition: Proof of a human choline requirement: A placebo-controlled trial. JPEN J. Parenter. Enteral Nutr..

[5] Zeisel S.H. (1981). Dietary choline: Biochemistry, physiology, and pharmacology. Annu. Rev. Nutr..

[6] Blusztajn J.K. (1998). Choline, a vital amine. Science.

[7] National Academies of Sciences, Engineering and Medicine (2021). Nutrient Requirements of Dairy Cattle.

[8] Kwan S.T.C., Ricketts D.K., Presswood B.H., Smith S.M., Mooney S.M. (2021). Prenatal choline supplementation during mouse pregnancy has differential effects in alcohol-exposed fetal organs. Alcohol Clin. Exp. Res..

[9] Ernst A.M., Gimbel B.A., de Water E., Eckerle J.K., Radke J.P., Georgieff M.K., Wozniak J.R. (2022). Prenatal and postnatal choline supplementation in fetal alcohol spectrum disorder. Nutrients.

[10] Yao Z., Vance D.E. (1990). Reduction in VLDL, but not HDL, in plasma of rats deficient in choline. Biochemistry Cell Biol..

[11] Fast D.G., Vance D.E. (1995). Nascent VLDL phospholipid composition is altered when phosphatidylcholine biosynthesis is inhibited: Evidence for a novel mechanism that regulates VLDL secretion. BBA-Lipids Lipid Metab..

[12] Emery R.S., Liesman J.S., Herdt T.H. (1992). Metabolism of long chain fatty acids by ruminant liver. J. Nutr..

[13] Bobe G., Young J.W., Beitz D.C. (2004). Invited review: Pathology, etiology, prevention, and treatment of fatty liver in dairy cows. J. Dairy Sci..

[14] Seymour D.J., Cánovas A., Baes C.F., Chud T.C.S., Osborne V.R., Cant J.P., Brito L.F., Gredler-Grandl B., Finocchiaro R., Veerkamp R.F. (2019). Invited review: Determination of large-scale individual dry matter intake phenotypes in dairy cattle. J. Dairy Sci..

[15] Drackley J.K. (1999). Biology of dairy cows during the transition period: The final frontier?. J. Dairy Sci..

[16] Duffield T. (2000). Subclinical ketosis in lactating dairy cattle. Vet. Clin. N. Am. Food Anim. Pract..

[17] Zom R.L., van Baal J., Goselink R.M., Bakker J.A., de Veth M.J., van Vuuren A.M. (2011). Effect of rumen-protected choline on performance, blood metabolites, and hepatic triacylglycerols of periparturient dairy cattle. J. Dairy Sci..

[18] Lima F.S., Sá Filho M.F., Greco L.F., Santos J.E.P. (2012). Effects of feeding rumen-protected choline on incidence of diseases and reproduction of dairy cows. Vet. J..

[19] Arshad U., Zenobi M.G., Staples C.R., Santos J.E.P. (2020). Meta-analysis of the effects of supplemental rumen-protected choline during the transition period on performance and health of parous dairy cows. J. Dairy Sci..

[20] Bollatti J.M., Zenobi M.G., Artusso N.A., Alfaro G.F., Lopez A.M., Barton B.A., Nelson C.D., Staples C.R., Santos J.E.P. (2020). Timing of initiation and duration of feeding rumen-protected choline affects performance of lactating Holstein cows. J. Dairy Sci..

[21] Wathes D.C., Fenwick M., Cheng Z., Bourne N., Llewellyn S., Morris D.G., Kenny D., Murphy J., Fitzpatrick R. (2007). Influence of negative energy balance on cyclicity and fertility in the high producing dairy cow. Theriogenology.

[22] White H.M. (2015). The role of TCA cycle anaplerosis in ketosis and fatty liver in periparturient dairy cows. Animals.

[23] Han van der Kolk J.H., Gross J.J., Gerber V., Bruckmaier R.M. (2017). Disturbed bovine mitochondrial lipid metabolism: A review. Vet. Q..

[24] Jia H., Li X., Liu G., Loor J.J., Bucktrout R., Sun X., Li G., Shu X., Dong J., Wang Y. (2019). Perilipin 5 promotes hepatic steatosis in dairy cows through increasing lipid synthesis and decreasing very low density lipoprotein assembly. J. Dairy Sci..

[25] Song Z., Xiaoli A.M., Yang F. (2018). Regulation and metabolic significance of de novo lipogenesis in adipose tissues. Nutrients.

[26] Planavila A., Iglesias R., Giralt M., Villarroya F. (2011). Sirt1 acts in association with PPARα to protect the heart from hypertrophy, metabolic dysregulation, and inflammation. Cardiovasc. Res..

[27] Mirza A.Z., Althagafi I.I., Shamshad H. (2019). Role of PPAR receptor in different diseases and their ligands: Physiological importance and clinical implications. Eur. J. Med. Chem..

[28] Lu D., He A., Tan M., Mrad M., El Daibani A., Hu D., Liu X., Kleiboeker B., Che T., Hsu F.F. (2024). Liver ACOX1 regulates levels of circulating lipids that promote metabolic health through adipose remodeling. Nat. Commun..

[29] Dong J., Loor J.J., Zuo R., Chen X., Liang Y., Wang Y., Shu X., Sun X., Jia H., Liu G. (2019). Low abundance of mitofusin 2 in dairy cows with moderate fatty liver is associated with alterations in hepatic lipid metabolism. J. Dairy Sci..

[30] Ding H., Li Y., Liu L., Hao N., Zou S., Jiang Q., Liang Y., Ma N., Feng S., Wang X. (2021). Sirtuin 1 is involved in oleic acid-induced calf hepatocyte steatosis via alterations in lipid metabolism-related proteins. J. Anim. Sci..

[31] Chamberlin W.G., Middleton J.R., Spain J.N., Johnson G.C., Ellersieck M.R., Pithua P. (2013). Subclinical hypocalcemia, plasma biochemical parameters, lipid metabolism, postpartum disease, and fertility in postparturient dairy cows. J. Dairy Sci..

[32] Yang W., Wang S., Loor J.J., Lopes M.G., Zhao Y., Ma X., Li M., Zhang B., Xu C. (2022). Role of diacylglycerol O-acyltransferase (DGAT) isoforms in bovine hepatic fatty acid metabolism. J. Dairy Sci..

[33] Zhou S., Chen M., Meng M., Ma N., Xie W., Shen X., Li Z., Chang G. (2023). Subclinical ketosis leads to lipid metabolism disorder by downregulating the expression of acetyl-coenzyme A acetyltransferase 2 in dairy cows. J. Dairy Sci..

[34] Liu L., Li X., Li Y., Guan Y., Song Y., Yin L., Chen H., Lei L., Liu J., Li X. (2014). Effects of nonesterified fatty acids on the synthesis and assembly of very low density lipoprotein in bovine hepatocytes in vitro. J. Dairy Sci..

[35] Yang W., Tian Y., Yang M., Mauck J., Loor J.J., Jia B., Wang S., Fan W., Li Z., Zhang B. (2024). β-sitosterol alleviates high fatty acid-induced lipid accumulation in calf hepatocytes by regulating cholesterol metabolism. J. Steroid Biochem. Mol. Biol..

[36] Li Y., Ding H., Liu L., Song Y., Du X., Feng S., Wang X., Li X., Wang Z., Li X. (2020). Non-esterified fatty acid induce dairy cow hepatocytes apoptosis via the mitochondria-mediated ROS-JNK/ERK signaling pathway. Front. Cell Dev. Biol..

[37] Wen Y., Wang Y., Loor J.J., Zhao C., Wang J. (2025). Non-esterified fatty acids disrupt hepatic lipid metabolism and mitochondrial function via TLR4/MyD88/IRAK2 signaling in bovine hepatocytes. J. Steroid Biochem. Mol. Biol..

[38] Zhu Y., Guan Y., Loor J.J., Sha X., Coleman D.N., Zhang C., Du X., Shi Z., Li X., Wang Z. (2019). Fatty acid-induced endoplasmic reticulum stress promoted lipid accumulation in calf hepatocytes, and endoplasmic reticulum stress existed in the liver of severe fatty liver cows. J. Dairy Sci..

[39] Lei L., Gao W., Loor J.J., Aboragah A., Fang Z., Du X., Zhang M., Song Y., Liu G., Li X. (2021). Reducing hepatic endoplasmic reticulum stress ameliorates the impairment in insulin signaling induced by high levels of β-hydroxybutyrate in bovine hepatocytes. J. Dairy Sci..

[40] Szegezdi E., Logue S.E., Gorman A.M., Samali A. (2006). Mediators of endoplasmic reticulum stress-induced apoptosis. EMBO Rep..

[41] Huang Y., Zhao C., Kong Y., Tan P., Liu S., Liu Y., Zeng F., Yuan Y., Zhao B., Wang J. (2021). Elucidation of the mechanism of NEFA-induced PERK-eIF2α signaling pathway regulation of lipid metabolism in bovine hepatocytes. J. Steroid Biochem. Mol. Biol..

[42] Reynolds C.K., Aikman P.C., Lupoli B., Humphries D.J., Beever D.E. (2003). Splanchnic metabolism of dairy cows during the transition from late gestation through early lactation. J. Dairy Sci..

[43] Zhang X., Huang C., Li X., Shangguan Z., Wei W., Liu S., Yang S., Liu Y. (2020). HFD and HFD-provoked hepatic hypoxia act as reciprocal causation for NAFLD via HIF-independent signaling. BMC Gastroenterol..

[44] Kong F., Lei L., Cai L., Li J., Zhao C., Liu M., Qi D., Gao J., Li E., Gao W. (2025). Hypoxia-inducible factor 2α mediates nonesterified fatty acids and hypoxia-induced lipid accumulation in bovine hepatocytes. J. Dairy Sci..

[45] Ren L.P., Yu X., Song G.Y., Zhang P., Sun L.N., Chen S.C., Hu Z.J., Zhang X.M. (2016). Impact of activating transcription factor 4 signaling on lipogenesis in HepG2 cells. Mol. Med. Rep..

[46] Dong J., Yue K., Loor J.J., Aboragah A., Li G., Chen L., Song Y., Du X., Liu G., Wang Z. (2022). Increased adipose tissue lipolysis in dairy cows with fatty liver is associated with enhanced autophagy activity. J. Dairy Sci..

[47] Contreras G.A., Strieder-Barboza C., Raphael W. (2017). Adipose tissue lipolysis and remodeling during the transition period of dairy cows. J. Anim. Sci. Biotechnol..

[48] Xu Q., Fan Y., Loor J.J., Liang Y., Sun X., Jia H., Zhao C., Xu C. (2021). Adenosine 5’-monophosphate-activated protein kinase ameliorates bovine adipocyte oxidative stress by inducing antioxidant responses and autophagy. J. Dairy Sci..

[49] Hao X., Liu M., Zhang X., Yu H., Fang Z., Gao X., Chen M., Shao Q., Gao W., Lei L. (2024). Thioredoxin-2 suppresses hydrogen peroxide-activated nuclear factor kappa B signaling via alleviating oxidative stress in bovine adipocytes. J. Dairy Sci..

[50] Pérez-Báez J., Risco C.A., Chebel R.C., Gomes G.C., Greco L.F., Tao S., Thompson I.M., do Amaral B.C., Zenobi M.G., Martinez N. (2019). Association of dry matter intake and energy balance prepartum and postpartum with health disorders postpartum: Part II. Ketosis and clinical mastitis. J. Dairy Sci..

[51] Suriyasathaporn W., Heuer C., Noordhuizen-Stassen E.N., Schukken Y.H. (2000). Hyperketonemia and the impairment of udder defense: A review. Vet. Res..

[52] Goulart D.B., Mellata M. (2022). *Escherichia coli* mastitis in dairy cattle: Etiology, diagnosis, and treatment challenges. Front. Microbiol..

[53] Hillreiner M., Flinspach C., Pfaffl M.W., Kliem H. (2016). Effect of the Ketone Body Beta-Hydroxybutyrate on the Innate Defense Capability of Primary Bovine Mammary Epithelial Cells. PLoS ONE.

[54] Khan M.Z., Li L., Wang T., Liu X., Chen W., Ma Q., Zahoor M., Wang C. (2024). Bioactive compounds and probiotics mitigate mastitis by targeting NF-κB signaling pathway. Biomolecules.

[55] Song Y., Loor J.J., Li C., Liang Y., Li N., Shu X., Yang Y., Feng X., Du X., Wang Z. (2021). Enhanced mitochondrial dysfunction and oxidative stress in the mammary gland of cows with clinical ketosis. J. Dairy Sci..

[56] Sun X., Chang R., Tang Y., Luo S., Jiang C., Jia H., Xu Q., Dong Z., Liang Y., Loor J.J. (2021). Transcription factor EB (TFEB)-mediated autophagy protects bovine mammary epithelial cells against H_2_O_2_-induced oxidative damage in vitro. J. Anim. Sci. Biotechnol..

[57] Mavangira V., Kuhn M.J., Abuelo A., Morisseau C., Hammock B.D., Sordillo L.M. (2021). Activity of sEH and oxidant status during systemic bovine coliform mastitis. Antioxidants.

[58] Chang R., Sun X., Jia H., Xu Q., Dong Z., Tang Y., Luo S., Jiang Q., Loor J.J., Xu C. (2022). Inhibiting nuclear factor erythroid 2 related factor 2-mediated autophagy in bovine mammary epithelial cells induces oxidative stress in response to exogenous fatty acids. J. Anim. Sci. Biotechnol..

[59] Yue K., Pu X., Loor J.J., Jiang Q., Dong J., Shen T., Li G., Gao W., Lei L., Du X. (2022). Impaired autophagy aggravates oxidative stress in mammary gland of dairy cows with clinical ketosis. J. Dairy Sci..

[60] Liu K., Dong Y., Cao L., Li G., Yang Z., Luo J., Lei L., Du X., Song Y., Usman M. (2025). Caveolin 1 ameliorates nonesterified fatty acid-induced oxidative stress via the autophagy regulator beclin 1 in bovine mammary gland epithelial cells. J. Dairy Sci..

[61] He Y., Su N., Yang H., Yang W., Zhao C., Fu Y., Hu Y., Hu X. (2023). ZEA mediates autophagy through the ROS-AMPK-m-TOR pathway to enhance the susceptibility of mastitis induced by Staphylococcus aureus in mice. Ecotoxicol. Environ. Saf..

[62] Shi Z., Li X.B., Peng Z.C., Fu S.P., Zhao C.X., Du X.L., Fang Z.Y., Wang Z., Liu G.W., Li X.W. (2018). Berberine protects against NEFA-induced impairment of mitochondrial respiratory chain function and insulin signaling in bovine hepatocytes. Int. J. Mol. Sci..

[63] Li P., Li L., Zhang C., Cheng X., Zhang Y., Guo Y., Long M., Yang S., He J. (2019). Palmitic acid and β-hydroxybutyrate induce inflammatory responses in bovine endometrial cells by activating oxidative stress-mediated NF-κB signaling. Molecules.

[64] Habib M.R., Tokutake Y., Yonekura S. (2025). Palmitic acid-induced cell death: Impact of endoplasmic reticulum and oxidative stress, mitigated by L-citrulline. Anim. Biosci..

[65] Hu H., Tian M., Ding C., Yu S. (2018). The C/EBP Homologous Protein (CHOP) Transcription Factor Functions in Endoplasmic Reticulum Stress-Induced Apoptosis and Microbial Infection. Front. Immunol..

[66] Zhu Q., Liu Z., Wang Y., Song E., Song Y. (2021). Endoplasmic reticulum stress manipulates autophagic response that antagonizes polybrominated diphenyl ethers quinone induced cytotoxicity in microglial BV2 cells. J. Hazard. Mater..

[67] Zhao C., Yu D., He Z., Bao L., Feng L., Chen L., Liu Z., Hu X., Zhang N., Wang T. (2021). Endoplasmic reticulum stress-mediated autophagy activation is involved in cadmium-induced ferroptosis of renal tubular epithelial cells. Free Radic. Biol. Med..

[68] Yan Y., Huang J., Huan C., Li L., Li C. (2022). Non-esterified fatty acid induces ER stress-mediated apoptosis via ROS/MAPK signaling pathway in bovine mammary epithelial cells. Metabolites.

[69] Chang R., Jia H., Dong Z., Xu Q., Liu L., Majigsuren Z., Batbaatar T., Xu C., Yang Q., Sun X. (2023). Free fatty acids induce apoptosis of mammary epithelial cells of ketotic dairy cows via the mito-ROS/NLRP3 signaling pathway. J. Agric. Food Chem..

[70] Krnjaić S., Cincović M., Djoković R., Belić B., Ježek J., Starič J. (2022). The influence of energy balance, lipolysis and ketogenesis on metabolic adaptation in cows milked twice and three times daily. Metabolites.

[71] Guadagnin A.R., Fehlberg L.K., Thomas B., Sugimoto Y., Shinzato I., Cardoso F.C. (2022). Effect of feeding rumen-protected lysine through the transition period on postpartum uterine health of dairy cows. J. Dairy Sci..

[72] Dai L., Liu Z., Guo L., Chai Y., Yang Y., Wang Y., Ma Y., Shi C., Zhang W. (2023). Multi-tissue transcriptome study of innate immune gene expression profiling reveals negative energy balance altered the defense and promoted system inflammation of dairy cows. Vet. Sci..

[73] Vanacker N., Hooper H.B., Blouin R., Lacasse P. (2023). Effect of intravenous lipid infusion on biomarkers of insulin resistance and immune functions of dry and nonpregnant dairy cows. J. Dairy Sci..

[74] Bogado Pascottini O., LeBlanc S.J. (2020). Metabolic markers for purulent vaginal discharge and subclinical endometritis in dairy cows. Theriogenology.

[75] Yáñez U., Herradón P.G., Becerra J.J., Peña A.I., Quintela L.A. (2022). Relationship between postpartum metabolic status and subclinical endometritis in dairy cattle. Animals.

[76] Scalia D., Lacetera N., Bernabucci U., Demeyere K., Duchateau L., Burvenich C. (2006). In vitro effects of nonesterified fatty acids on bovine neutrophils oxidative burst and viability. J. Dairy Sci..

[77] Wagener K., Gabler C., Drillich M. (2017). A review of the ongoing discussion about definition, diagnosis and pathomechanism of subclinical endometritis in dairy cows. Theriogenology.

[78] Ingvartsen K.L., Moyes K.M. (2015). Factors contributing to immunosuppression in the dairy cow during the periparturient period. Jpn. J. Vet. Res..

[79] LeBlanc S.J. (2014). Reproductive tract inflammatory disease in postpartum dairy cows. Animal.

[80] Seifi H.A., Dalir-Naghadeh B., Farzaneh N., Mohri M., Gorji-Dooz M. (2007). Metabolic changes in cows with or without retained fetal membranes in transition period. J. Vet. Med. A Physiol. Pathol. Clin. Med..

[81] Swangchan-Uthai T., Chen Q., Kirton S.E., Fenwick M.A., Cheng Z., Patton J., Fouladi-Nashta A.A., Wathes D.C. (2013). Influence of energy balance on the antimicrobial peptides S100A8 and S100A9 in the endometrium of the post-partum dairy cow. Reproduction.

[82] Li K., Zhou P., Li J., Cheng Y., Li S., Wang Y., Jiang W., Bai Y., Cao H., Wang D. (2023). Upregulation of P2Y14 receptor in neutrophils promotes inflammation after myocardial ischemia/reperfusion injury. Life Sci..

[83] Wu N., Zhang J., Chen Y., Xu Q., Song P., Li Y., Li K., Liu H. (2022). Recent advances in microbial production of L-malic acid. Appl. Microbiol. Biotechnol..

[84] Li Q., Cao Z., Ling X., Sun P., Yin W., Fan K., Sun N., Li H. (2024). Potential molecular targets and pathways of a traditional Chinese medicine formula for bovine endometritis identified by network pharmacology. Pol. J. Vet. Sci..

[85] Chankeaw W., Guo Y.Z., Båge R., Svensson A., Andersson G., Humblot P. (2018). Elevated non-esterified fatty acids impair survival and promote lipid accumulation and pro-inflammatory cytokine production in bovine endometrial epithelial cells. Reprod. Fertil. Dev..

[86] Ferst J.G., Glanzner W.G., Gutierrez K., de Macedo M.P., Ferreira R., Gasperin B.G., Duggavathi R., Gonçalves P.B., Bordignon V. (2021). Supplementation of oleic acid, stearic acid, palmitic acid and β-hydroxybutyrate increase H3K9me3 in endometrial epithelial cells of cattle cultured in vitro. Anim. Reprod. Sci..

[87] Pell J.M., Bates P.C. (1990). The nutritional regulation of growth hormone action. Nutr. Res. Rev..

[88] Wathes D.C., Cheng Z., Fenwick M.A., Fitzpatrick R., Patton J. (2011). Influence of energy balance on the somatotrophic axis and matrix metalloproteinase expression in the endometrium of the postpartum dairy cow. Reproduction.

[89] Aungier S.P., Roche J.F., Diskin M.G., Crowe M.A. (2014). Risk factors that affect reproductive target achievement in fertile dairy cows. J. Dairy Sci..

[90] Beltman M.E., McNally J.C., Kelly E., Crowe M.A. (2020). Relationship between plasma concentrations of IGF-I and clinical endometritis, and response to progesterone synchrony in dairy cows during early lactation. J. Dairy Sci..

[91] Tufarelli V., Puvača N., Glamočić D., Pugliese G., Colonna M.A. (2024). The most important metabolic diseases in dairy cattle during the transition period. Animals.

[92] Zhang H., Clemens R.A., Liu F., Hu Y., Baba Y., Theodore P., Kurosaki T., Lowell C.A. (2014). STIM1 calcium sensor is required for activation of the phagocyte oxidase during inflammation and host defense. Blood.

[93] Elling R., Keller B., Weidinger C., Häffner M., Deshmukh S.D., Zee I., Speckmann C., Ehl S., Schwarz K., Feske S. (2016). Preserved effector functions of human ORAI1- and STIM1-deficient neutrophils. J. Allergy Clin. Immunol..

[94] Zhang B., Guo H., Yang W., Li M., Zou Y., Loor J.J., Xia C., Xu C. (2019). Effects of ORAI calcium release-activated calcium modulator 1 (ORAI1) on neutrophil activity in dairy cows with subclinical hypocalcemia1. J. Anim. Sci..

[95] Bréchard S., Tschirhart E.J. (2008). Regulation of superoxide production in neutrophils: Role of calcium influx. J. Leukoc. Biol..

[96] Noubade R., Wong K., Ota N., Rutz S., Eidenschenk C., Valdez P.A., Ding J., Peng I., Sebrell A., Caplazi P. (2014). NRROS negatively regulates reactive oxygen species during host defence and autoimmunity. Nature.

[97] Curtis C.R., Erb H.N., Sniffen C.J., Smith R.D., Powers P.A., Smith M.C., White M.E., Hillman R.B., Pearson E.J. (1983). Association of parturient hypocalcemia with eight periparturient disorders in Holstein cows. J. Am. Vet. Med. Assoc..

[98] Melendez P., Donovan G.A., Risco C.A., Goff J.P. (2004). Plasma mineral and energy metabolite concentrations in dairy cows fed an anionic prepartum diet that did or did not have retained fetal membranes after parturition. Am. J. Vet. Res..

[99] Sharma B.K., Erdman R.A. (1989). In Vitro Degradation of choline from selected foodstuffs and choline supplements. J. Dairy Sci..

[100] Arruda A.G., Godden S., Rapnicki P., Gorden P., Timms L., Aly S.S., Lehenbauer T.W., Champagne J. (2013). Randomized noninferiority clinical trial evaluating 3 commercial dry cow mastitis preparations: I. Quarter-level outcomes. J. Dairy Sci..

[101] Sharma B.K., Erdman R.A. (1989). Effects of dietary and abomasally infused choline on milk production responses of lactating dairy cows. J. Nutr..

[102] Holdorf H.T., Kendall S.J., Ruh K.E., Caputo M.J., Combs G.J., Henisz S.J., Brown W.E., Bresolin T., Ferreira R.E.P., Dorea J.R.R. (2023). Increasing the prepartum dose of rumen-protected choline: Effects on milk production and metabolism in high-producing Holstein dairy cows. J. Dairy Sci..

[103] Sales J., Homolka P., Koukolova V. (2010). Effect of dietary rumen-protected choline on milk production of dairy cows: A meta-analysis. J. Dairy Sci..

[104] Zenobi M.G., Gardinal R., Zuniga J.E., Dias A.L.G., Nelson C.D., Driver J.P., Barton B.A., Santos J.E.P., Staples C.R. (2018). Effects of supplementation with ruminally protected choline on performance of multiparous Holstein cows did not depend upon prepartum caloric intake. J. Dairy Sci..

[105] Shahsavari A., Michael J.D., Al Jassim R. (2016). The role of rumen-protected choline in hepatic function and performance of transition dairy cows. Br. J. Nutr..

[106] Davidson S., Hopkins B.A., Odle J., Brownie C., Fellner V., Whitlow L.W. (2008). Supplementing limited methionine diets with rumen-protected methionine, betaine, and choline in early lactation Holstein cows. J. Dairy Sci..

[107] McFadden J.W., Girard C.L., Tao S., Zhou Z., Bernard J.K., Duplessis M., White H.M. (2020). Symposium review: One-carbon metabolism and methyl donor nutrition in the dairy cow. J. Dairy Sci..

[108] Pinotti L., Campagnoli A., D’ambrosio F., Susca F., Innocenti M., Rebucci R., Fusi E., Cheli F., Savoini G., Dell’Orto V. (2008). Rumen-protected choline and vitamin E supplementation in periparturient dairy goats: Effects on milk production and folate, vitamin B12 and vitamin E status. Animal.

[109] Mečionytė I., Palubinskas G., Anskienė L., Japertienė R., Juodžentytė R., Žilaitis V. (2022). The effect of supplementation of rumen-protected choline on reproductive and productive performances of dairy cows. Animals.

[110] Petersson K.J., Strandberg E., Gustafsson H., Berglund B. (2006). Environmental effects on progesterone profile measures of dairy cow fertility. Anim. Reprod. Sci..

[111] Adriaens I., Martin O., Saeys W., De Ketelaere B., Friggens N.C., Aernouts B. (2019). Validation of a novel milk progesterone-based tool to monitor luteolysis in dairy cows: Timing of the alerts and robustness against missing values. J. Dairy Sci..

[112] Ardalan M., Rezayazdi K., Dehghan-Banadaky M. (2010). Effect of rumen-protected choline and methionine on physiological and metabolic disorders and reproductive indices of dairy cows. J. Anim. Physiol. Anim. Nutr..

[113] Morrison E.I., Reinhardt H., Leclerc H., DeVries T.J., LeBlanc S.J. (2018). Effect of rumen-protected B vitamins and choline supplementation on health, production, and reproduction in transition dairy cows. J. Dairy Sci..

[114] Acosta D.A.V., Rivelli M.I., Skenandore C., Zhou Z., Keisler D.H., Luchini D., Corrêa M.N., Cardoso F.C. (2017). Effects of rumen-protected methionine and choline supplementation on steroidogenic potential of the first postpartum dominant follicle and expression of immune mediators in Holstein cows. Theriogenology.

[115] Bellato A., Tondo A., Dellepiane L., Dondo A., Mannelli A., Bergagna S. (2023). Estimates of dairy herd health indicators of mastitis, ketosis, inter-calving interval, and fresh cow replacement in the Piedmont region, Italy. Prev. Vet. Med..

[116] Ren X., Lu H., Wang Y., Yan L., Liu C., Chu C., Yang Z., Bao X., Yu M., Zhang Z. (2024). Phenotypic and genetic analyses of mastitis, endometritis, and ketosis on milk production and reproduction traits in Chinese holstein cattle. Animals.

[117] Tsiamadis V., Banos G., Panousis N., Kritsepi-Konstantinou M., Arsenos G., Valergakis G.E. (2016). Genetic parameters of calcium, phosphorus, magnesium, and potassium serum concentrations during the first 8 days after calving in Holstein cows. J. Dairy Sci..

[118] Marques T.C., Monteiro H.F., Melo D.B., Coelho W.M., Salman S., Marques L.R., Leão K.M., Machado V.S., Menta P., Dubey D. (2024). Effect of rumen-protected choline on dairy cow metabolism, immunity, lactation performance, and vaginal discharge microbiome. J. Dairy Sci..

[119] Furken C., Hoedemaker M. (2014). Influence of feeding rumen-protected choline to transition dairy cows. Part 2: Health and reproduction. Tierarztl. Prax. Ausg. G Grosstiere Nutztiere.

[120] Bollatti J.M., Zenobi M.G., Artusso N.A., Lopez A.M., Nelson C.D., Barton B.A., Staples C.R., Santos J.E.P. (2020). Effects of rumen-protected choline on the inflammatory and metabolic status and health of dairy cows during the transition period. J. Dairy Sci..

[121] Goselink R.M.A., Van Baal J., Widjaja H.C.A., Dekker R.A., Zom R.L.G., De Veth M.J., Van Vuuren A.M. (2013). Effect of rumen-protected choline supplementation on liver and adipose gene expression during the transition period in dairy cattle. J. Dairy Sci..

[122] Arshad U., Husnain A., Poindexter M.B., Zimpel R., Perdomo M.C., Santos J.E.P. (2023). Effect of source and amount of rumen-protected choline on hepatic metabolism during induction of fatty liver in dairy cows. J. Dairy Sci..

[123] Gluchowski N.L., Becuwe M., Walther T.C., Farese R.V. (2017). Lipid droplets and liver disease: From basic biology to clinical implications. Nat. Rev. Gastroenterol. Hepatol..

[124] Schulze R.J., Drižytė K., Casey C.A., McNiven M.A. (2017). Hepatic lipophagy: New insights into autophagic catabolism of lipid droplets in the liver. Hepatol. Commun..

[125] Arshad U., Husnain A., Poindexter M.B., Zimpel R., Nelson C.D., Santos J.E.P. (2023). Rumen-protected choline reduces hepatic lipidosis by increasing hepatic triacylglycerol-rich lipoprotein secretion in dairy cows. J. Dairy Sci..

[126] Artegoitia V.M., Middleton J.L., Harte F.M., Campagna S.R., De Veth M.J. (2014). Choline and choline metabolite patterns and associations in blood and milk during lactation in dairy cows. PLoS ONE.

[127] De Veth M.J., Artegoitia V.M., Campagna S.R., Lapierre H., Harte F., Girard C.L. (2016). Choline absorption and evaluation of bioavailability markers when supplementing choline to lactating dairy cows. J. Dairy Sci..

[128] Arshad U., Zenobi M.G., Tribulo P., Staples C.R., Santos J.E.P. (2023). Dose-dependent effects of rumen-protected choline on hepatic metabolism during induction of fatty liver in dry pregnant dairy cows. PLoS ONE.

[129] Shen J., Sun B., Yu C., Cao Y., Cai C., Yao J. (2020). Choline and methionine regulate lipid metabolism via the AMPK signaling pathway in hepatocytes exposed to high concentrations of nonesterified fatty acids. J. Cell. Biochem..

[130] Chandler T.L., White H.M. (2017). Choline and methionine differentially alter methyl carbon metabolism in bovine neonatal hepatocytes. PLoS ONE.

[131] Morello E., Sutti S., Foglia B., Novo E., Cannito S., Bocca C., Rajsky M., Bruzzì S., Abate M.L., Rosso C. (2018). Hypoxia-inducible factor 2α drives nonalcoholic fatty liver progression by triggering hepatocyte release of histidine-rich glycoprotein. Hepatology.

[132] Sun F., Cao Y., Cai C., Li S., Yu C., Yao J. (2016). Regulation of nutritional metabolism in transition dairy cows: Energy homeostasis and health in response to post-ruminal choline and methionine. PLoS ONE.

[133] Zenobi M.G., Gardinal R., Zuniga J.E., Mamedova L.K., Driver J.P., Barton B.A., Santos J.E.P., Staples C.R., Nelson C.D. (2020). Effect of prepartum energy intake and supplementation with ruminally protected choline on innate and adaptive immunity of multiparous Holstein cows. J. Dairy Sci..

[134] Lopreiato V., Vailati-Riboni M., Bellingeri A., Khan I., Farina G., Parys C., Loor J.J. (2019). Inflammation and oxidative stress transcription profiles due to in vitro supply of methionine with or without choline in unstimulated blood polymorphonuclear leukocytes from lactating Holstein cows. J. Dairy Sci..

[135] Villa R.F., Ferrari F., Gorini A. (2012). Effect of CDP-choline on age-dependent modifications of energy- and glutamate-linked enzyme activities in synaptic and non-synaptic mitochondria from rat cerebral cortex. Neurochem. Int..

[136] Yang M., Kuang M., Wang G., Ali I., Tang Y., Yang C., Li Y., Li L. (2021). Choline attenuates heat stress-induced oxidative injury and apoptosis in bovine mammary epithelial cells by modulating PERK/Nrf-2 signaling pathway. Mol. Immunol..

